# Bacterial Acid Resistance Toward Organic Weak Acid Revealed by RNA-Seq Transcriptomic Analysis in *Acetobacter pasteurianus*

**DOI:** 10.3389/fmicb.2019.01616

**Published:** 2019-08-06

**Authors:** Haoran Yang, Yongjian Yu, Caixia Fu, Fusheng Chen

**Affiliations:** ^1^Hubei International Scientific and Technological Cooperation Base of Traditional Fermented Foods, College of Food Science and Technology, Huazhong Agricultural University, Wuhan, China; ^2^Jiangsu Hengshun Vinegar Industry Co., Ltd., Zhenjiang, China; ^3^Hubei Tulaohan Flavouring and Food Co., Ltd., Yichang, China

**Keywords:** acetic acid bacteria, *Acetobacter pasteurianus*, acid resistance, acetic acid, RNA-Seq transcriptome

## Abstract

Under extreme acidic environments, bacteria exploit several acid resistance (AR) mechanisms for enhancing their survival, which is concerned with several aspects, such as issues in human health and fermentation for acidic products. Currently, knowledge of bacterial AR mainly comes from the strong acid (such as hydrochloric acid) stresses, whereas AR mechanisms against organic weak acids (such as acetic acid), which are indeed encountered by bacteria, are less understood. Acetic acid bacteria (AAB), with the ability to produce acetic acid up to 20 g/100 mL, possess outstanding acetic acid tolerance, which is conferred by their unique AR mechanisms, including pyrroloquinoline quinine-dependent alcohol dehydrogenase, acetic acid assimilation and molecular chaperons. The distinguished AR of AAB toward acetic acid may provide a paradigm for research in bacterial AR against weak organic acids. In order to understand AAB’s AR mechanism more holistically, omics approaches have been employed in the corresponding field. However, the currently reported transcriptomic study was processed under a low-acidity (1 g/100 mL) environment, which could not reflect the general conditions that AAB are usually faced with. This study performed RNA-Seq transcriptomic analysis investigating AR mechanisms in *Acetobacter pasteurianus* CGMCC 1.41, a widely used vinegar-brewing AAB strain, at different stages of fermentation, namely, under different acetic acid concentrations (from 0.6 to 6.03 g/100 mL). The results demonstrated the even and clustered genomic distribution of up- and down-regulated genes, respectively. Difference in AR between AAB and other microorganisms was supported by the down-regulation of urea degradation and trehalose synthesis-related genes in response to acetic acid. Detailed analysis reflected the role of ethanol respiration as the main energy source and the limited effect of acetic acid assimilation on AR during fermentation as well as the competition between ethanol respiratory chain and NADH, succinate dehydrogenase-based common respiratory chain. Molecular chaperons contribute to AR, too, but their regulatory mechanisms require further investigation. Moreover, pathways of glucose catabolism and fatty acid biosynthesis are also related to AR. Finally, 2-methylcitrate cycle was proposed as an AR mechanism in AAB for the first time. This study provides new insight into AR mechanisms of AAB, and it also indicates the existence of numerous undiscovered AR mechanisms.

## Introduction

Extreme acidic environments are of great challenge to bacteria, as pH homeostasis is crucial for their living (Krulwich et al., 2011). Thus, bacteria that usually encounter such environments have to evolve several mechanisms to enhance survival (Liu et al., 2015). For example, it is well known that gastric juice has extremely low pH due to the presence of hydrochloric acid (HCl), forming a barrier blocking pathogenic microbes and probiotics (Foster, 2004). However, some bacteria such as *Escherichia coli* and lactic acid bacteria (LAB) are able to pass through the stomach in relatively lower doses thanks to their acid resistance (AR) (Lin et al., 1996; Foster, 2004; Kanjee and Houry, 2013; Hlaing et al., 2018). Faced with such a low pH environment, these bacteria exploit several AR mechanisms including the glutamic acid–dependent acid resistance (GDAR) system, F_1_–F_0_–ATPase proton pump, biofilm formation, protection or repair of macromolecules and alkali production (Kanjee and Houry, 2013; Lund et al., 2014; Liu et al., 2015). The proton-consuming GDAR, which involves glutamate decarboxylase and glutamate/γ-aminobutyric acid antiporter, is considered the dominant AR mechanism under extremely low pH conditions (pH from 2.0 to 3.0) (Foster, 2004; De Biase and Pennacchietti, 2012; Kanjee and Houry, 2013). In addition, glutamine can be converted to glutamate by protein YbaS with release of ammonia, hence it supports GDAR and consequently provides robust AR to bacterial cells (Lu et al., 2013).

Besides inorganic strong acid such as HCl, bacterial cells also suffer from organic weak acids. In the intestinal track, they will encounter stress from short-chain fatty acids—in particular, acetate, propionate, and butyrate (Koh et al., 2016)—which are produced from microbiota-accessible carbohydrates (Sonnenburg and Sonnenburg, 2014). Meanwhile, recombinant protein-producing *E. coli* strains also generate acetate as one of the byproducts that causes stress (Eiteman and Altman, 2006). Since carboxylic weak acids hardly dissociate, their undissociated form can penetrate into and subsequently acidify the cytoplasm in a much easier way than strong acids do (Lund et al., 2014; Trcek et al., 2015). Therefore, weak acids not only bring about low pH environment, but also force cells to deal with the entire acid molecules instead of only protons, which might be different from AR against strong acids. However, efficient bacterial resistance toward organic weak acids is less understood compared to that for strong acids.

Acetic acid bacteria (AAB), especially *Acetobacter* and *Komagataeibacter* strains, possess outstanding abilities to tolerate as well as produce acetic acid, a kind of organic weak acid, hence are mainly used for vinegar production (Gullo et al., 2014; Saichana et al., 2015; Wang et al., 2015a). AAB strains can produce at most 20 g/100 mL of acetic acid from incomplete oxidation of ethanol (Sokollek et al., 1998), while 0.5 g/100 mL of acetic acid will impose distinct inhibition or even a lethal impact on most microorganisms (Conner and Kotrola, 1995). The distinguished acetic acid tolerance of AAB suggests that deciphering AAB AR mechanisms may provide a paradigm for studies in stress response of bacterial cells facing the organic weak acids. Meanwhile, knowledge on AAB AR mechanisms is also required for improving vinegar production (Xia et al., 2016).

Therefore, AR mechanisms in AAB have become the focus of research, and several unique AR mechanisms have been revealed. For example, acetic acid can be assimilated through a specialized tricarboxylic acid (TCA) cycle, where the prevalent succinyl-CoA synthetase is replaced by AarC, functioning as succinyl-CoA: acetate CoA transferase (Mullins et al., 2008; Azuma et al., 2009), or it can be expelled out of the cytoplasm by a proton motive force-dependent efflux system (Matsushita et al., 2005) and an ATP-binding cassette (ABC) transporter AatA (Nakano et al., 2006). Moreover, acetic acid-producing related enzyme in AAB cells, pyrroloquinoline quinine (PQQ)-dependent alcohol dehydrogenase (ADH), has been found to be closely related to AR of AAB (Trcek et al., 2006; Wang et al., 2015b), while changes in membrane composition (Trcek et al., 2007) and molecular chaperons (Okamoto-Kainuma and Ishikawa, 2016) are also involved in AR of AAB.

In order to holistically understand AR mechanisms in AAB, genomics and proteomics studies have been conducted for exploring integral AR systems against acetic acid, which have been reviewed previously (Nakano and Fukaya, 2008; Wang et al., 2015a; Xia et al., 2017). Nevertheless, genomic research cannot provide the dynamic gene expression data under different conditions (i.e., at different acidity), while as the expression of genes are reflected both on transcription and translation levels, proteomic studies can only provide expression profiles on translation level. Gene expression profiles on transcription level provided by transcriptomic analysis are also required for a better understanding of AR mechanisms in AAB. Unfortunately, transcriptome approaches are rarely applied in this field. To our knowledge, there is only one DNA-microarray-based transcriptomic study concerning the AR of AAB, with the maximum acidity (denoted as acetic acid concentration) reached only about 1 g/100 mL (Sakurai et al., 2012), which could not reflect the general circumstances that AAB are usually faced with, as they can produce acetic acid far more than 1 g/100 mL (Sokollek et al., 1998). The absence of transcriptomic studies regarding AR mechanisms under higher acidity conditions may block a comprehensive understanding in AR regulatory strategies.

In this study, RNA-Seq transcriptomic analysis was performed to investigate the AR in *A. pasteurianus* CGMCC 1.41, an AAB strain widely used in China for brewing vinegar. The strain was inoculated in Treatment Medium (TM) I and II to perform acetic acid fermentation. Both TM I and TM II were sampled at three points, together with a control sample collected from a non-acetic acid-producing CK medium. This study aims to reveal expression patterns of AR-related genes under different stages of fermentation, namely, under conditions with different acetic acid concentrations at the transcriptional level, which will help improve understanding of AAB AR mechanisms.

## Materials and Methods

### Strains Cultivation and Fermentation

In the present study, CK medium refers to GYP (1 g/L of glucose, 5 g/L of yeast extract and 2 g/L of peptone) medium; TM I and TM II refer to GYP medium with 3% ethanol and 6% ethanol with the addition of 0.5% acetic acid, respectively. *A. pasteurianus* CGMCC 1.41 was purchased from China General Microbiological Culture Collection Center. Strain stored in glycerol cube was activated and trained by inoculating in 50 mL GYP medium with 3% ethanol contained in a 250 mL Erlenmeyer flask. When the exponential phase (OD_600__nm_ between 0.6 and 0.8) was reached, 5 mL of the culture was then inoculated in 50 mL CK and TM I media contained in the 250 mL Erlenmeyer flask. When the newly inoculated culture in TM I reached the exponential phase, 5 mL of this culture was inoculated in 50 mL TM II contained in the 250 mL Erlenmeyer flask. Cultivation was conducted under shaking at 170 rpm and 30°C. The acidity of the culture was measured by titration using 0.1 M NaOH with phenolphthalein as indicator.

### RNA Extraction

The extraction of RNA samples was performed by the method used by Sakurai et al. (2012) with a little modification. When the sampling points were reached, 2 mL of the culture was centrifugated at 10000 rpm for 3 min, the supernatant was discarded followed by suspending the pellet with another 500 μL culture. The concentrated cultures were stored at -80°C for 12–24 h, and then 1 mL of RNAprotect Bacteria Reagent (Qiagen) was immediately added as soon as the concentrated cultures were completely melted at room temperature. After incubating for 5–10 min at room temperature, the mixtures were centrifugated at 5000 *g* for 10 min at room temperature, then the supernatant was discarded. Unless the temperature was additionally mentioned, all of the following operations were manipulated on ice. 200 μL of 15 mg/mL lysozyme solved in TE buffer (pH 8.0) together with 20 μL 10% SDS solution were added to the pellet, the resulting suspension was subsequently incubated at 65°C for 1–2 min. Then, 20 μL of 1 M sodium acetate (pH 5.2) along with 1 mL water-saturated acidic phenol were added followed by incubating at 65°C for 6 min with inversion every 1 min. Afterward, the mixture was immediately centrifugated at 12000 rpm and 4°C for 10 min. The acquired aqueous layer was mixed with an equal volume of phenol/chloroform (1:1), with vigorous shaking for 30 s followed by immediate centrifugation at 12000 rpm and 4°C for 10 min. The acquired aqueous layer was mixed with an equal volume of chloroform with vigorous shaking for 30 s. Soon, the mixture was then centrifugated at 12000 rpm and 4°C for 10 min. The acquired aqueous layer was mixed with 1/10 volume of 3 M sodium acetate (pH 5.2), 1/10 volume of 1 mM EDTA solution and 2.5× volume of ice-cold ethanol. The mixture was stored at -80°C for 0.5–1 h, followed by centrifugation at 13000 rpm and 4°C for 30 min. After discarding the supernatant, the pellet was washed twice by adding 1 mL 75% ice-cold ethanol and centrifugation at 7500 rpm and 4°C for 5 min. The ethanol was completely discarded, followed by solving the pellet in 50 μL diethyl pyrocarbonate (DEPC)-treated water. The quality of the extracted RNA was checked by Agilent 2100. Prior to being used for RNA extraction, all of the tools and solutions (except for lysozyme solution prepared with DEPC-treated TE buffer) were treated with 0.1% DEPC solution by shaking at 37°C for at least 12 h, and then sterilized.

### Sequencing and Analysis

#### RNA-Seq Transcriptome Resequencing, Raw Data Filtering and Clean Reads Mapping

After the removal of the rRNA and the fragmentation of mRNA, the cDNA library was constructed and then subjected to Illumina HiSeq 2000 sequencing. The sequencing quality of each base within the reads was represented as a value sQ, which was calculated by the formula:

$$\mathrm{sQ}=-10\times \mathrm{lg}\left(\frac{E}{1-E}\right)/(\mathrm{lg10)}$$

where E represents the sequencing error rate. The raw data were filtered by removal of the reads with sequence adaptors, or with “N” base >10%, or with low quality (bases with an sQ value less than or equal to 10 accounts for more than 50% of the entire read) to obtain clean reads. Clean reads were then mapped to the genome of *A. pasteurianus* CGMCC 1.41, which was sequenced by our lab in a previous work (Wang et al., 2015b). Mapping was carried out by Bowtie software.

#### Quantification of Gene Expression, and Differently Expressed Genes (DEGs) Identification

The expression level of genes was shown as Fragments Per Kilobase Million (FPKM) value, which was calculated by the formula below:

$$\mathrm{FPKM}=\frac{10^{6}C}{\mathrm{NL}/10^{3}}$$

in which C is the number of fragments uniquely mapped to a specified gene, N is the number of fragments uniquely mapped to all the predicted genes and L is the length of the specified gene. The DEGs were identified by the Poisson distribution-based method (Audic and Claverie, 1997) with the following criteria: fold change ≥2 and false discovery rate (FDR) ≤0.001. GO annotation was conducted with Blast2GO software^1^ with default parameters. The GO enrichment analysis was performed on OmicShare platform^2^, terms with FDR ≤0.05 were regarded as significantly enriched terms.

#### Data Visualization

The genome-wide map of Log_2_ (fold change) value of DEGs among the treatment samples was constructed by Circos 0.69 software (Krzywinski et al., 2009). The clustering of gene expression patterns and the corresponding heatmap were performed and constructed by MeV 4.0 (Saeed et al., 2003) software using Euclidean Distance.

### Data Availability

Sequencing data of this study have been deposited to Sequence Read Archive (SRA)^3^, with the project number PRJNA549106.

## Results

### Overview and the General Information of RNA-Seq Transcriptome of *A. pasteurianus* CGMCC 1.41

**FIGURE 1:**
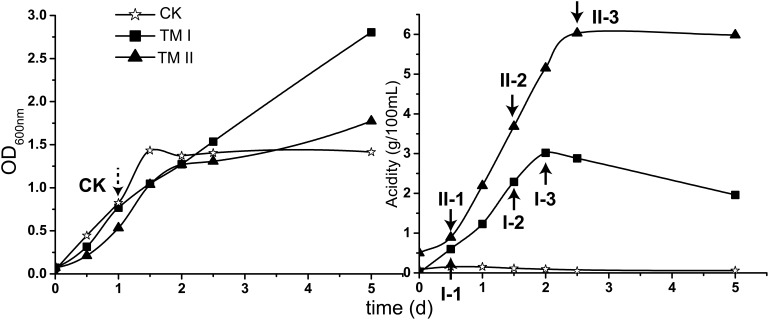
Growth (shown in OD_600__nm_ value) and acidity of the culture together with the sampling point in this study. CK sample was collected when the culture in CK medium reached exponential phase (dash-lined arrow); the treatment samples were collected when the acetic acid fermentation entered initial, mid and final stages in TM I (I-1, I-2, and I-3) and TM II (II-1, II-2, and II-3) media, respectively (solid-lined arrow).

For carrying out this study, *A. pasteurianus* CGMCC 1.41 was cultured in CK, TM I and TM II media, respectively. Profiles of growth (shown in OD_600__nm_) and acidity in each culture are shown in Figure 1.

During the cultivation period within this study, cells in CK medium experienced exponential and stationary phases. The exponential phase was reached at the 1st day of cultivation when the OD_600__nm_ value reached around 0.8 (Figure 1). The culture in TM I presented consecutive growth throughout the cultivation, with the decreasing in acidity after reaching its maximum of 3.02 g/100 mL at the 2nd day of fermentation. Differently, acidity in TM II continuously increased within 2 days and a half and maintained its maximum value of 6.03 g/100 mL (Figure 1).

For RNA-Seq sampling, the treatment samples were collected when the fermentation in both TM I and TM II entered initial (I-1: acidity of 0.60 g/100 mL; II-1: acidity of 0.89 g/100 mL), mid (I-2: acidity of 2.29 g/100 mL; II-2: acidity of 3.68 g/100 mL), and final (I-3: acidity of 3.02 g/100 mL; II-3: acidity of 6.03 g/100 mL) stages, respectively (Figure 1, solid-lined arrows). The CK sample was collected when the culture reached the exponential phase (Figure 1, dash-lined arrow).

**FIGURE 2:**
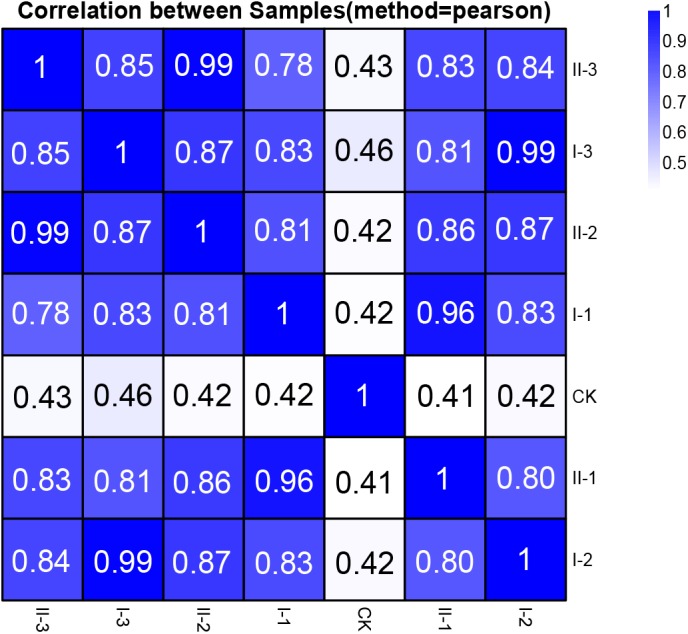
Pearson correlation value of gene expression between samples of this study. The correlation value between each sample was calculated based on the FPKM value of all the genes. Each row and column of the figure both represent samples, the number and the square color below represent the correlation value between pair of samples. The more two samples are similar with each other, the closer the correlation value is to 1.

The extracted RNA of all the samples was the subject of the RNA-Seq transcriptome sequencing. The acquired reads passed the quality control, and subsequently were utilized for genome mapping and gene expression qualification for further analysis. The correlation analysis based on the integral gene expression quantification of each sample (Figure 2) demonstrated that, CK was obviously distinguished from all the treatment samples, while gene expression profiles are relatively similar among treatment samples (Pearson correlation value ≥0.78). It is worth noting that expression profiles of the samples collected from the mid and final stages of fermentation within the same media (I-2 vs. I-3; II-2 vs. II-3) are almost identical (Pearson correlation value 0.99), as well as samples collected from the initial stage of fermentation within different media (I-1 vs. II-1, Pearson correlation value 0.96) (Figure 2). It is suggested that similar strategies might be exploited to initiate fermentation regardless of the difference in media content, while biological activities may be nearly unchanged at the mid and final stages of the fermentation, whereas the content in different media will bring differences in gene expression profiles.

Compared to CK, 1558, 1604, 1575, 1508, 1502, and 1428 DEGs were identified in samples I-1, I-2, I-3, II-1, II-2, and II-3, respectively, approximately accounting for 50% of genes within the genome. In addition, most of DEGs were up-regulated, accounting for about 40% of the genome (Table 1). The Gene Ontology (GO) enrichment analysis (Supplementary Table S1) revealed that, the up-regulated genes mainly encode intracellular proteins, while products of the down-regulated genes are mainly located on membrane. Meanwhile, the up-regulated genes mainly conduct RNA binding functions and are involved in several metabolic processes, such as organonitrogen compound and small molecule metabolic process; while the down-regulated ones mainly perform respiration-related functions, such as oxidoreductase and NADH dehydrogenase activities, and are mainly involved in processes related to respiration and energy derivation.

**Table 1:** Statics of DEGs of treatment samples.

Sample	No. of up-regulated genes	No. of down-regulated genes	Total number of DEGs
I-1	1254 (42%)^a^	304 (10%)	1558 (52%)
I-2	1320 (44%)	284 (9%)	1604 (53%)
I-3	1296 (43%)	279 (9%)	1575 (52%)
II-1	1181 (39%)	327 (11%)	1508 (50%)
II-2	1202 (40%)	300 (10%)	1502 (50%)
II-3	1142 (38%)	286 (9%)	1428 (47%)


*^a^Percentages shown in the brackets indicate the proportion that accounts for total genes within the genome.*

**FIGURE 3:**
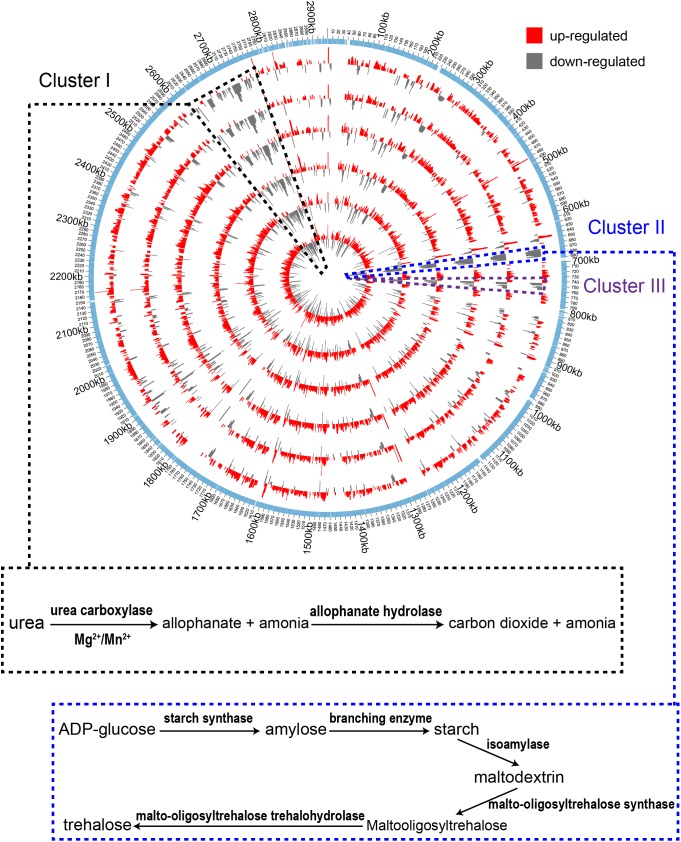
Genome-wide map of Log_2_ (fold change) value of DEGs among the treatment samples. From inner to outer, the circles successively represent samples I-1, II-1, I-2, I-3, II-2, and II-3 (with increase in acidity of the cultures). The outmost circle shows the protein-coding genes within the genome. The up-regulated genes are shown to distribute throughout the genome. The down-regulated genes were mainly clustered in three regions within the genome (Cluster I, II, and III) indicated by the black, blue and dark purple dashed lines, respectively. Identified pathways encoded by genes within the clustered down-regulated regions are shown in the box connected to the corresponding area where the genes are located.

In order to understand gene expression profiles at the genome scale, Log_2_ (fold change) values of DEGs of the treatment samples were mapped to the genome of *A. pasteurianus* CGMCC 1.41 using Circos 0.69 software (Krzywinski et al., 2009). As shown in Figure 3, the up-regulated genes were found evenly distributed throughout the entire genome, while interestingly, the down-regulated genes, even much fewer than the up-regulated ones, were mainly clustered in three genomic regions (Cluster I, II, and III, Figure 3). Genes residing in these clusters were further investigated. However, it is hard to decipher the relationship with AR of all the down-regulated genes within these regions at once, but some clues were also discovered. It is revealed that Cluster I harbors genes related to urea amidolyase activity, which processes the degradation of urea to ammonia and carbon dioxide (Lin et al., 2016), while Cluster II covers an operon responsible for synthesizing trehalose from maltooligosaccharides (Maruta et al., 1995; Figure 3 and Supplementary Table S2). The cleavage of urea into ammonia and carbon dioxide acts as an AR mechanism in other bacteria species, thanks to the proton neutralizing ability of ammonia (Liu et al., 2015), while trehalose is considered as being able to protect bacterial and yeast cells from multiple stresses (Argüelles, 2000; Nicolaou et al., 2010). Additionally, expression of genes AS.899–AS.905, encoding the urease system of *A. pasteurianus* CGMCC 1.41, was nearly unchanged during fermentation (Supplementary Table S2). Taken together, these results indicated that ammonia production via urea degradation and trehalose synthesis may not contribute to AR in AAB, suggesting the difference in AR between AAB and other microorganisms.

### Detailed Analysis of Gene Expression Related to AR in AAB

In order to obtain insight into AR mechanisms in AAB, detailed analysis of expression profiles of AR-related genes was performed. PQQ-ADH and PQQ- aldehyde dehydrogenase (ALDH) catalyze the incomplete oxidation of ethanol for producing acetic acid (Saichana et al., 2015; Wang et al., 2015a). Located on the periplasm side of the cell membrane with ubiquinone oxidoreductase activity, the two enzymes form a typical ethanol respiratory chain in AAB, coupling the ATP-generating oxidative phosphorylation, so that AAB cells can directly utilize the energy released from incomplete oxidation of ethanol (Matsushita et al., 2004). Additionally, AAB possess components of respiratory chain prevalent in other species, that is, NADH dehydrogenase complex (complex I) and succinate dehydrogenase complex (complex II), which are referred to as common respiratory chain in this study. In addition to producing acetic acid, PQQ-ADH is involved in AR as well (Trcek et al., 2006; Wang et al., 2015b). Meanwhile, acetic acid assimilation and TCA cycle (Fukaya et al., 1990, 1993; Nakano et al., 2004; Mullins et al., 2008; Nakano and Fukaya, 2008; Wang et al., 2015a; Andres-Barrao et al., 2016), molecular chaperons (Okamoto-Kainuma and Ishikawa, 2016) and acetic acid pumping-out systems (Matsushita et al., 2005; Nakano et al., 2006) are also proposed as AAB AR mechanisms, as mentioned in the Introduction section. Moreover, observation of membrane composition changes in AAB under acetic acid stress conditions (Trcek et al., 2007) may suggest the importance of membrane in their AR mechanisms, which may further imply the involvement of fatty acid biosynthesis in AR systems, as phospholipids are the major components of the membrane.

In the following paragraphs, expression profiles of genes related to ethanol and common respiratory chains, acetic acid assimilation and glucose catabolism will be presented from the view of AR and energy metabolism. In addition, expression patterns of genes regarding fatty acid biosynthesis, acetic acid transporter and molecular chaperons, which are also related to AR, will be displayed as well. Finally, a novel potential AR mechanism of AAB, 2-methylcitrate cycle, will be demonstrated.

#### Ethanol Oxidation and Respiratory Chain

**FIGURE 4:**
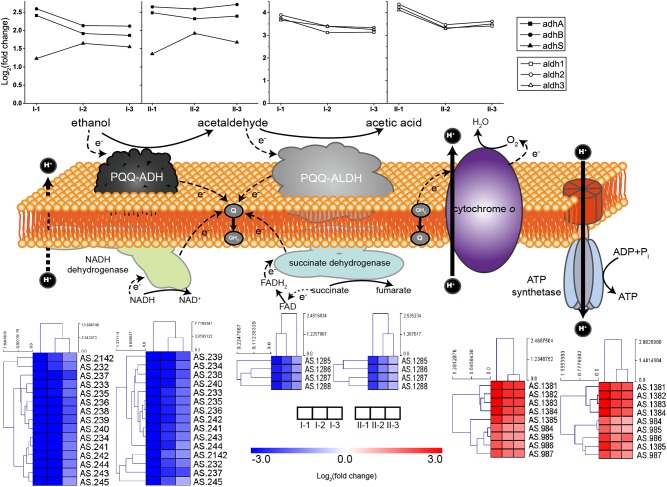
Illustration and expression patterns of genes for ethanol and common respiratory chains, as well as genes coding for ATP synthetase. During oxidation of ethanol to acetic acid, both of the membranes bound PQQ-ADH and PQQ-ALDH transfer electrons to ubiquinone (Q), the reducing form (QH_2_) of which is subsequently oxidized by terminal oxidase cytochrome O with pumping out the protons to generate proton motive force (PMF). PMF is then utilized by ATP synthetase for ATP generation. Therefore, PQQ-ADH and PQQ-ALDH form a typical ethanol respiratory chain in AAB, making AAB cells able to directly and rapidly utilize the energy released from incomplete oxidation of ethanol. The expression profiles of genes are placed right above (for ethanol respiratory chain, i.e., PQQ-ADH and PQQ-ALDH, in curves) or below (for common respiratory chain, i.e., NADH dehydrogenase and succinate dehydrogenase, and ATP synthetase, in heatmaps) the corresponding components. Detailed FPKM and Log_2_ (fold change) values together with the annotation of each gene are listed in Supplementary Table S2.

Both PQQ-ADH and PQQ-ALDH are three-subunit protein complexes. Genes coding for the three subunits of PQQ-ALDH are clustered in the genome, while the small subunit gene *adhS* of PQQ-ADH is separated from *adhA-adhB* operon encoding the two large subunits (Kondo et al., 1995; Illeghems et al., 2013). Subunits of AdhA and AdhB play a key role in ethanol-ubiquinone oxidoreductase activity, while AdhS acts as a molecular chaperon of AdhA and is not considered as directly participating in ethanol oxidation (Yakushi and Matsushita, 2010). The respiratory chains together with the expression profile of corresponding genes are illustrated in Figure 4. Compared to CK, genes encoding the ethanol respiratory chain (PQQ-ADH and PQQ-ALDH) were significantly up-regulated. In contrast, those coding for the common respiratory chain were significantly down-regulated. Meanwhile, ATP synthetase genes were also up-regulated during fermentation (Figure 4), emphasizing that ATP is mainly generated via ethanol respiratory chain and thus ethanol is the main energy source under acetic acid-producing conditions. Notably, decreasing in degree of both down-regulation of genes for common respiratory chain and up-regulation of those encoding PQQ-ALDH in both TM I and TM II, together with *adhA* and *adhB* in TM I, was observed at the mid and final stages of the fermentation (Figure 4), suggesting a competition between the two respiratory chains. However, *adhA* and *adhB* retained high expression levels all along in TM II (Figure 4), suggesting the importance of PQQ-ADH in dealing with higher acidity.

#### Acetic Acid Assimilation (Overoxidation) and TCA Cycle

**FIGURE 5:**
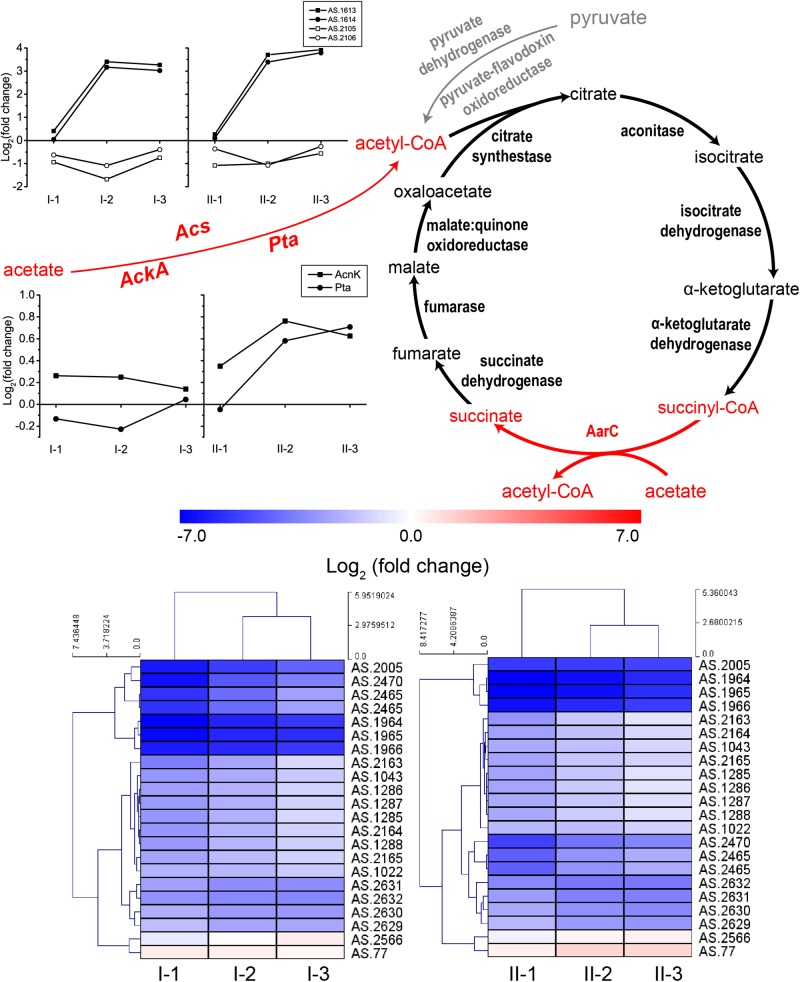
Illustration of acetic acid assimilation and expression profiles of genes related to acetic acid assimilation: TCA cycle (down, in heatmap), *Acs* (up, in curves), as well as *AckA* and *Pta* (middle, in curves). Acetic acid is assimilated through the specialized TCA cycle, where the prevalent succinyl-CoA synthetase is replaced by AarC, succinyl-CoA: acetate CoA transferase, which convert succinyl-CoA to succinate in exchange of converting acetic acid to acetyl-CoA (shown in red). The assimilation starts with the transformation of acetic acid to acetyl-CoA, which is shown in red in the figure. Detailed FPKM and Log_2_ (fold change) values together with the annotation of each gene are listed in Supplementary Table S2.

Acetic acid needs to be converted into acetyl-CoA prior to being assimilated through the specialized TCA cycle. The conversion can be conducted by protein AarC. In addition, acetyl-CoA synthase (Acs), as well as acetate kinase (AckA) and phosphotransacetylase (Pta), can also carry out this process (Wang et al., 2015a). The acetic acid assimilation process together with the expression profiles of the corresponding genes are shown in Figure 5. Since this process not only directly eliminates acetic acid molecules, but also supplies energy, all the genes involved in acetic acid assimilation were estimated to be significantly up-regulated during fermentation. Unexpectedly, however, in this study, they were not. Genes encoding enzymes involved in TCA cycle were even significantly down-regulated compared to CK, while the expression profile of *Acs, AckA*, and *Pta* was not significantly changed during the fermentation process, except for two *Acs* genes AS.1613 and AS.1614 (Figure 5). However, the two genes are assumed to actually encode propionyl-CoA synthase, thus they are related to 2-methylcitrate cycle (see below). Therefore, acetic acid assimilation was demonstrated to be suppressed during the ethanol oxidation process. On the other hand, most of the TCA cycle enzymes coding genes exhibited a decreased degree in down-regulations as the fermentation went by (Figure 5), the same expression pattern as genes of the common respiratory chain, which means these genes were up-regulated at the mid and final stages of the fermentation compared to the initial stage. This suggested that, along with the decline in ethanol concentration during the fermentation process, the specialized TCA cycle together with the common respiratory chain might gradually work to oxidize acetic acid for energy supply (stoichiometrically, 4.23 g/100 mL and 8.57 g/100 mL of acetic acid will be produced in TM I and TM II, respectively, but actually, as shown in Figure 1, only 3.02 g/100 mL and 6.03 g/100 mL were produced, which indicated the oxidation of part of the acetic acid during fermentation).

#### Glucose Catabolism and Fatty Acid Biosynthesis

**FIGURE 6:**
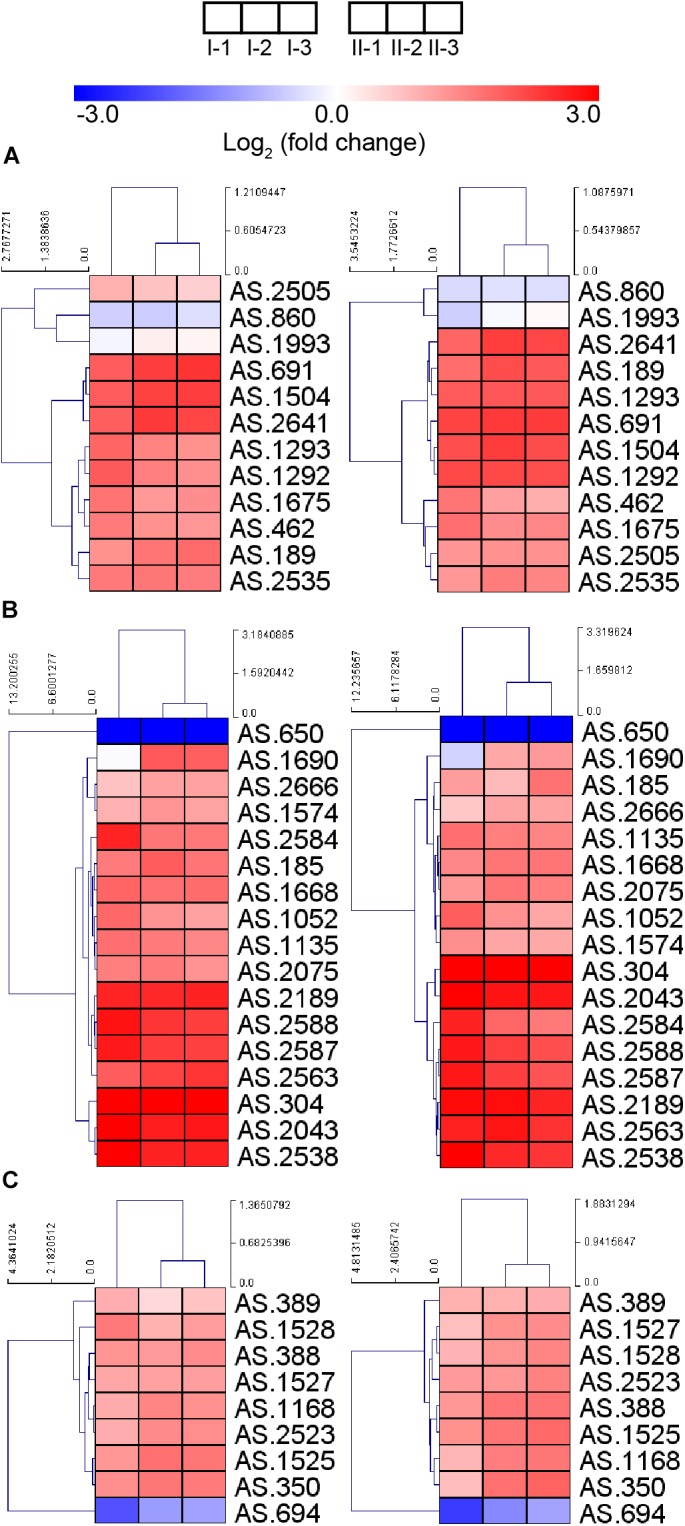
Heatmap of expression profiles of genes related to **(A)** glycolysis and oxidative phase of PPP, **(B)** fatty acid biosynthesis, and **(C)** molecular chaperons. Detailed FPKM and Log_2_ (fold change) value, together with the annotation of each gene, are listed in Supplementary Table S2.

Due to the up-regulation of ethanol respiratory chain and down-regulation of common respiratory chain as well as the TCA cycle, glucose was considered as losing its position as the main energy source during acetic acid fermentation. However, most genes involved in the glucose-catabolizing glycolysis pathway and the oxidative phase of the pentose phosphate pathway (PPP) were up-regulated under fermentation conditions (Figure 6A). This result may be in line with a previous study, where glucose was shown to be consumed during acetic acid fermentation (Zheng et al., 2017). The consumption of glucose might be in favor of producing several intermediate metabolites or reducing power (NADPH) generation, which is required for anabolic process (such as fatty acid biosynthesis), while it may have little role in supplying energy.

Genes involved in fatty acid biosynthesis, except AS.650 encoding a short chain dehydrogenase FabG, were also up-regulated during the fermentation process (Figure 6B), indicating the importance of fatty acid in stress response under fermentation conditions. It is further implied that the enhanced fatty acid biosynthesis may contribute a lot to lipid content of the cell wall and membrane, thus strengthening their integrity within the adverse environment, which may be required during the acetic acid producing process (Wang et al., 2015c). Nevertheless, the decreased up-regulation of gene *fabD* (AS.2588) and several *fabG* genes (AS.1688, AS.1690, AS.2075, and AS.2666) at the mid and final stages of fermentation was observed, indicating they were down-regulated compared to the initial stage of fermentation, which was consistent with a previous proteomic research reporting the down-regulation of FabD and FabG proteins in response to the higher acidity at later period of fermentation (Xia et al., 2016).

#### Acetic Acid Pump-Out and Molecular Chaperons

Bacterial cells may be able to pump out chemical substances that will bring harmful effect. In AAB, a proton motive force-dependent efflux system (Matsushita et al., 2005) and an ABC transporter AatA (Nakano et al., 2006) have been reported to be responsible for pumping out acetic acid. Unfortunately, the structure and the composition of the proton motive force-dependent efflux system are still unknown, making the investigation of expression patterns of the system inaccessible. Meanwhile, gene *aatA* (AS.2689) encoding AatA did not show obvious change in expression in this study (Supplementary Table S2), which was in line with the observation from the previous transcriptomic study in *Acetobacter aceti* (Sakurai et al., 2012). Besides the pumping-out systems, molecular chaperons are proposed to provide AAB protection against stress during fermentation process too (Okamoto-Kainuma and Ishikawa, 2016). In this study, genes encoding several molecular chaperons, including GroES, GroEL, DnaK, DnaJ, GrpE, and ClpB, were up-regulated (Figure 6C), suggesting the requirement of assistance from molecular chaperons to correctly fold and repair the damaged proteins under fermentation conditions. Unexpectedly, gene AS.694 encoding the reported regulator of these molecular chaperons RpoH (Okamoto-Kainuma et al., 2011) was down-regulated during fermentation (Figure 6C), indicating the elaborate transcription regulation of molecular chaperon genes, which requires further investigations.

#### Potential Novel AR Mechanism: 2-Methylcitate Cycle

**FIGURE 7:**
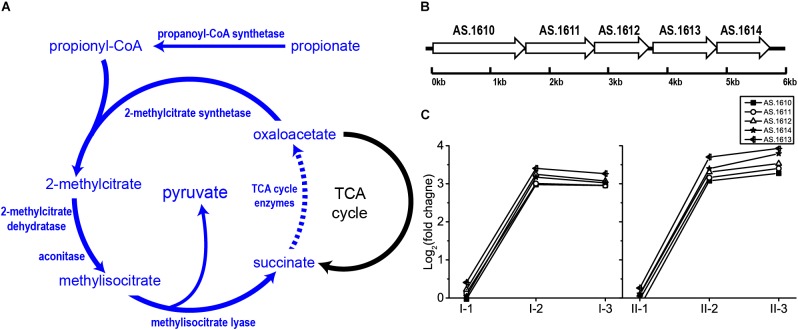
Illustration of **(A)** 2-methylcitrate cycle pathway and **(B)** genomic arrangement of genes within operon AS.1610–1614 in *A. pasteurianus* CGMCC 1.41 (homolog to *prpBCDE* operon in *Salmonella* spp.) encoding 2-methylcitrate cycle enzymes besides TCA cycle, as well as **(C)** their expression profiles. Detailed FPKM and Log_2_ (fold change) values together with the annotation of each gene are listed in Supplementary Table S2.

Originally, 2-methylcitrate cycle is considered as a propionate metabolism pathway, as reported in *E. coli* (Brock et al., 2002), *Salmonella* spp. (Horswill and Escalante-Semerena, 2001) and *Bacillus subtilis* (Reddick et al., 2017). This pathway begins with the formation of propionyl-CoA from propionate conducted by propionyl-CoA synthetase. Subsequently, 2-methylcitrate synthetase catalyzes the junction of propionyl-CoA and oxaloacetate to form 2-methylcitrate, which will be converted into methylisocitrate by 2-methylcitrate dehydratase and aconitase. Then, methylisocitrate is cleaved into succinate and pyruvate catalyzed by methylisocitrate lyase. Oxaloacetate is subsequently regained from succinate through TCA cycle, which initiates a new round of 2-methylcitrate cycle (Horswill and Escalante-Semerena, 2001; Reddick et al., 2017; Figure 7). In *Salmonella* spp., all enzymes of 2-methylcitrate cycle besides TCA cycle are encoded by *prpBCDE* operon (Horswill and Escalante-Semerena, 2001), which is homologous to operon AS.1610–AS.1614 in *A. pasteurianus* CGMCC 1.41. Being within the 2-methylcitrate cycle-related operon, AS.1613 and AS.1614 were therefore assumed to be a single gene encoding propionyl-CoA synthase rather than Acs. In this study, genes within operon AS.1610–AS.1614 were significantly up-regulated in the mid and final stages of the fermentation in both TM I and TM II (Figure 7). Meanwhile, as mentioned before, when compared to the initial stage of fermentation, genes within TCA cycle were up-regulated at the mid and final stages. Hence, these results reflect the potential contribution of 2-methylcitrate cycle to AR in AAB. To our knowledge, this is the first time of reporting the involvement of 2-methylcitrate cycle in AR strategy of AAB.

## Discussion

Due to outstanding tolerance toward acetic acid, AAB AR mechanisms will add valuable knowledge in the encyclopedia of bacterial AR strategies, especially stress response for organic weak acid. Owing to the different chemical properties of inorganic strong acid and organic weak acid, differences between AR systems against these two kinds of acids may exist. Actually, the robust AR provider GDAR is absent in AAB, while pathways for urea degradation and trehalose synthesis in *A. pasteurianus* CGMCC 1.41, which are reported related to bacterial AR and stress response, respectively, were down-regulated after acetic acid was produced, as demonstrated in this study. Like AAB, LAB are well known to produce organic acid, lactic acid. However, LAB possess several AR mechanisms that are similar with those in *E. coli* (Wang et al., 2017), which are vigorous for resisting inorganic strong acids. Indeed, being one of the probiotics in the gastrointestinal track, LAB also encounter stress from gastric juice. On the other hand, these AR mechanisms may be less effective for counteracting carboxylic acid. For example, for free lactic acid production from LAB, neutralizing agents such as calcium carbonate are required for promoting lactic acid yield, which may reflect that LAB strains are still sensitive to organic week acids even they possess AR mechanisms (Liu et al., 2015; Singhvi et al., 2018). In addition, production of lactic acid at acidic environments without neutralizing agents can be achieved by constructing protoplast fusion using *A. pasteurianus* and *Lactobacillus delbrueckii* Uc-3 (Singhvi et al., 2015), suggesting the robust protection against organic acid could be provided by distinguishing AR systems in AAB.

It has been revealed that AAB do possess unique AR mechanisms. The specialized TCA cycle-dependent acetic acid assimilation is such an example. However, because that large amount of acetic acid is truly accumulated in the culture during acetic acid fermentation, the efficiency of this process is rationally limited at the time, as its suppression is the guarantee for acetic acid accumulation. Moreover, the down-regulation of TCA cycle also blocks the glucose oxidation for energy supply. In contrast, genes of the ethanol respiratory chain were up-regulated during fermentation. Therefore, the rapid incomplete oxidation of ethanol through the ethanol respiratory chain becomes the main energy source for AAB cells during fermentation. However, despite that the oxidoreductase part of PQQ-ADH in TM II retained a high expression level during the whole fermentation process, competition between common and ethanol respiratory chains was indicated by a decreased degree in both up-regulation of ethanol respiratory chain and down-regulation of common respiratory chain (Figure 4), as well as the TCA cycle (Figure 5), which supplies NADH for electron transferring. In a previous study, the competition of respiratory chains was also revealed in another AAB strain, *Gluconbacter oxydans* DSM3504 (Kostner et al., 2015). As ethanol is being consumed for acetic acid production, the common respiratory chain, together with the TCA cycle, might gradually take the place of ethanol respiratory chain in energy generation, facilitating the overoxidation of a small fraction of acetic acid at the same time, which can alleviate the stress from acetic acid to some extent. Therefore, cells need to keep a delicate balance between acetic acid accumulation and assimilation during the fermentation process. Meanwhile, consistent high expression of *adhA* and *adhB* in TM II may emphasize the importance of PQQ-ADH in AR systems of AAB. Unfortunately, the relationship between PQQ-ADH and AR is still unclear. However, due to the broad substrate specificity (Yakushi and Matsushita, 2010), PQQ-ADH may be involved in oxidation of other substrates when the culture has high acetic acid and low ethanol concentration, providing energy for other AR mechanisms within the cells (Matsushita et al., 2004; Trcek et al., 2015).

Expelling acetic acid is another AR strategy in AAB. Up to now, the ABC transporter AatA is the only characterized transporter responsible for pumping out acetic acid. However, the gene encoding this transporter, *aatA*, did not obviously up-regulate during fermentation, as was also observed in previous transcriptomic research (Sakurai et al., 2012). This phenomenon can be explained from several aspects. First, AatA was induced and identified through a proteomic approach (Nakano et al., 2006), thus post-transcription regulation may exist for gene *aatA*. On the other hand, in the aforementioned proteomic study, acetic acid was manually added to the culture instead of being produced from ethanol by AAB cells themselves. Hence, it can be speculated that acetic acid may have fewer opportunities to penetrate into the AAB cells when ethanol is present, making AatA less required for expelling acetic acid during fermentation. Reduced infiltration of acetic acid might be the consequence of the strengthened cell membrane and its lowered permeability, probably supported by the enhanced fatty acid biosynthesis. This is consistent with the down-regulation of acetic acid assimilation, especially at the initial stage of fermentation, as this process is conducted intracellularly. Nevertheless, as a result of the decreased down-regulation of TCA cycle and the oxidation of small fraction of acetic acid, as well as the decreased up-regulation of fatty acid biosynthesis-related *fabD* and *fabG* genes, acetic acid may have more chances to enter the cells at the later stages of fermentation, where the concentration of ethanol is decreased while that of acetic acid is increased. Therefore, it can be further assumed that membrane permeability for acetic acid might depend on the ratio of ethanol/acetic acid concentration, i.e., the lower the ratio, the higher the acetic acid permeability.

Propionate metabolism related 2-methylcitrate cycle may contribute to AR in AAB when the acidity reaches the relatively high-level during fermentation. The cleavage of methylisocitrate within 2-methylcitrate cycle is analog to the plausible AR-conferring glyoxylate pathway in *A. aceti*, where isocitrate is split into succinate and glyoxylate by isocitrate lyase (Sakurai et al., 2013; Wang et al., 2015a). In the previous transcriptomic study, genes of the glyoxylate pathway were up-regulated when cells were oxidizing ethanol (Sakurai et al., 2012), which is similar to the expression pattern of operon AS.1610–AS.1614 in the present study (Figure 7). *A. pasteurianus* CGMCC 1.41 does not possess genes of the glyoxylate pathway but harbors those of 2-methylcitrate cycle. It is therefore very interesting to investigate the relationship between the two potential AR mechanisms in AAB which conduct analogous reactions with their genes exhibiting similar expression patterns in further studies. In addition, the role of 2-methylcitrate cycle in AR of AAB may lie in supplying pyruvate and succinate. Pyruvate is a well-known important intersection of metabolic pathways, and was also reported to be related to AR in bacteria (Wu et al., 2014). Meanwhile, adding succinate to the culture has been proven to be effective for enhancing acetic acid fermentation (Qi et al., 2013). However, since this is a newly proposed AR-conferring pathway in AAB, some important questions remain. For example, the connection of propionate metabolism to AR, as well as the source of propionate pool during acetic acid fermentation is unclear, while the detailed roles of succinate and pyruvate in AR are yet to be investigated. Hence, the relationship between 2-methylcitrate cycle and AAB AR systems requires further exploration.

In addition to 2-methylcitrate cycle, numerous novel AR mechanisms in AAB are yet to be discovered, as approximately 40% of the genes within the genome were up-regulated during fermentation, whereas the relationship between many of them and AR has not been elucidated. Previous proteomic study also suggested the involvement of the whole cellular system for dealing with the acid stress (Xia et al., 2016). The undiscovered AR mechanisms are likely to contain regulatory strategies for gene expression, which are currently poorly characterized in AAB. In *E. coli*, global regulator RpoS is closely related to its AR (Foster, 2004; Bak et al., 2014), while engineering of other global regulators such as H-NS (Gao et al., 2018) and RpoD (Gao et al., 2016) also improves acid tolerance. Additionally, several other regulatory mechanisms related to AR in *E. coli* were proposed (Foster, 2004). Therefore, interesting findings will likely be acquired from future studies concerning AAB regulatory networks behind their AR mechanisms.

In this study, transcriptomic analysis regarding AR in AAB was conducted with the maximum acidity as high as 6 g/100 mL (sample II-3, Figure 1) for the first time, providing a new insight into AR mechanisms against acetic acid. On the other hand, owing to the difficulties in RNA extraction under acidic conditions, currently we are unable to carry out transcriptomic study under conditions with higher concentration of acetic acid. Xia et al. (2016) suggested that cells may exhibit distinct biological process when the acidity is higher than 7% (approximately equal to 7 g/100 mL). Thus, new RNA extraction strategies may be required for transcriptomic studies under more severe acidic conditions. In further studies, comparison of acid stress responses between higher (acidity >7 g/100 mL) and mild acidic conditions may lead to more interesting findings concerning bacterial AR for counteracting organic carboxylic acid; meanwhile, for a deeper understanding of AR mechanisms, effective marker-less gene deletion and other molecular manipulation systems would be necessary in such high-acid-producing AAB strains for further investigating the findings from the omics research.

## Author Contributions

FC supervised the entire work and planned the experiments. HY performed the experiments, analyses, and wrote the manuscript. YY, CF, and FC revised the manuscript.

## Conflict of Interest Statement

YY was employed by company Jiangsu Hengshun Vinegar Industry Co., Ltd. CF was employed by company Hubei Tulaohan Flavouring and Food Co., Ltd. The remaining authors declare that the research was conducted in the absence of any commercial or financial relationships that could be construed as a potential conflict of interest.

## Footnotes

1 https://www.blast2go.com/

2 http://www.omicshare.com/tools/

3 https://www.ncbi.nlm.nih.gov/sra/
